# Supplementary material for: Antimicrobial resistant gene prevalence in soils due to animal manure deposition and long-term pasture management
Source: PeerJ. 2020 Nov 3;8:e10258. doi: 10.7717/peerj.10258 (PMC7646296; doi:10.7717/peerj.10258)
Supplement: Supplemental Information 1 [file peerj-08-10258-s001.docx]

Supplementary Table 1.

| **Class** | **CG** | **H** | **RBR** | **RBS** | **Resistance Mechanism** |
| --- | --- | --- | --- | --- | --- |
| Multi-drug resistance | 58 | 30 | 47 | 41 | Multi-drug efflux pumps |
|  | 11 | 1 | 9 | 6 | MDR regulator |
|  | 1 | 1 | 3 | 0 | MDR mutant porin proteins |
| Betalactams | 20 | 9 | 20 | 9 | Class A betalactamases |
|  | 7 | 3 | 15 | 5 | Class C betalactamases |
|  | 14 | 5 | 7 | 5 | Class B betalactamases |
|  | 10 | 1 | 7 | 6 | Class D betalactamases |
|  | 4 |  | 2 | 0 | Mutant porin proteins |
|  | 2 |  |  |  | Penicillin binding protein |
| MLS | 16 | 3 | 25 | 11 | 23S rRNA methyltransferases |
|  | 1 |  | 5 | 4 | ABC transporter |
|  | 2 | 2 | 3 | 2 | Macrolide resistance efflux pumps |
|  | 6 | 4 | 4 | 3 | Macrolide glycosyltransferases |
|  |  |  | 1 |  | Macrolide esterases |
|  | 2 | 2 | 1 | 1 | Macrolide phosphotransferases |
|  | 2 |  | 1 |  | Spiramycin efflux pumps |
|  |  |  | 0 | 1 | Lincosamide nucleotidyltransferases |
|  | 1 |  |  |  | Streptogramin B ester bond cleavage |
|  | 1 | 2 |  |  | Streptogramin resistance ATP-binding cassette ABC efflux pumps |
| Aminoglycosides | 1 |  | 1 | 2 | 16S rRNA methyltransferases |
|  | 2 | 2 | 1 | 3 | Aminoglycoside O-nucleotidyltransferases |
|  |  |  | 1 | 0 | Aminoglycoside-resistant 30S ribosomal subunit protein S12 |
|  | 10 | 3 | 18 | 10 | Aminoglycoside N-acetyltransferases |
|  | 11 | 4 | 12 | 6 | Aminoglycoside O-phosphotransferases |
|  | 1 |  |  |  | Aminoglycoside-resistance regulator mutation |
|  | 1 | 1 |  |  | Aminoglycoside-resistant 16S-23S rRNA O-methyltransferases |
| Tetracyclines | 13 | 8 | 20 | 5 | Tetracycline resistance major facilitator superfamily MFS efflux pumps |
|  | 4 | 1 | 6 | 3 | Tetracycline transcriptional repressor |
|  | 2 | 2 | 3 | 1 | Tetracycline resistance ribosomal protection proteins |
| Rifampin | 22 | 13 | 12 | 9 | Rifampin-resistant beta-subunit of RNA polymerase RpoB |
|  | 2 | 2 | 2 | 2 | Rifampin phosphotransferase |
|  | 2 | 1 | 2 | 0 | Monooxygenase |
|  | 0 | 2 | 1 | 1 | Rifampin ADP-ribosyltransferase Arr |
|  | 0 |  | 1 | 1 | Rifampin glycosyltransferase |
|  | 1 |  |  |  | RNA-polymerase binding protein |
| Fluoroquinolones | 18 | 9 | 16 | 18 | Fluoroquinolone-resistant DNA topoisomerases |
|  | 1 | 1 |  |  | Quinolone active efflux |
|  | 2 | 1 |  |  | Quinolone resistance protein Qnr |
| Mycobacterium tuberculosis-specific Drug | 1 |  | 7 | 1 | Ethambutol resistant arabinosyltransferase |
|  | 2 |  | 1 | 0 | Isoniazid-resistant kasA |
|  | 4 |  | 1 | 1 | Viomycin phosphotransferases |
|  |  | 1 | 1 | 0 | Ethionamide-resistant monooxygenase EthA |
|  | 2 | 1 | 2 | 0 | Ethambutol-resistant iniC |
|  | 2 |  | 0 | 1 | Isoniazid-resistant inhA |
|  | 2 | 1 |  |  | Isoniazid-resistant ndh |
| Glycopeptides | 5 | 2 | 6 | 2 | VanA-type resistance protein |
|  | 1 |  |  |  | VanA-type regulator |
|  |  |  | 1 | 0 | Undecaprenol pyrophosphate recycling |
|  | Supplementary Table 1. Continued | | | | |
|  |  |  | 1 | 0 | VanA-type accessory protein |
|  |  |  | 1 | 0 | VanC-type resistance protein |
|  | 1 |  | 1 | 0 | VanD-type regulator |
|  |  |  | 1 | 0 | VanE-type resistance protein |
|  | 1 |  |  |  | VanB-type resistance protein |
|  | 1 | 1 |  |  | VanI-type accessory protein |
|  | 2 | 1 |  |  | VanO-type regulator |
|  |  |  |  | 1 | Bleomycin resistance protein |
|  |  | 1 |  |  | VanC-type regulator |
|  |  | 1 |  |  | VanG-type accessory protein |
| Aminocoumarins | 8 |  | 8 | 3 | Aminocoumarin-resistant DNA topoisomerases |
|  | 3 | 2 | 2 | 1 | Aminocoumarin efflux pump |
| Phenicol | 3 | 3 | 5 | 7 | Phenicol efflux pumps |
|  |  |  | 2 | 1 | Chloramphenicol acetyltransferases |
| Elfamycins | 3 | 5 | 6 | 6 | EF-Tu inhibition |
| Cationic Antibioitic peptides | 1 | 1 | 4 | 2 | Lipid A modification |
|  |  | 2 |  | 1 | Polymyxin B resistance regulator |
| Fosfomycin |  | 1 | 3 | 3 | Fosfomycin phosphorylation |
|  | 1 |  | 1 |  | Fosfomycin thiol transferases |
| Sulfonamides | 10 |  | 4 | 4 | Sulfonamide-resistant dihydropteroate synthases |
| Fusidic acid | 2 |  | 3 | 1 | Fusidic acid esterases |
| Metronidazole |  |  | 2 |  | nim nitroimidazole reductase |
| Thiostrepton |  |  | 2 |  | Thiostrepton 23S rRNA methyltransferases |
| Trimethoprim | 2 |  | 1 | 2 | Dihydrofolate reductase |
| Bacitracin | 1 |  |  |  | Bacitracin ABC transporter |

Supplementary Table 2. Antibiotics and vaccines used on cattle in grazing treatments from 2004 - 2016 in Booneville, AR.

| **Date** | **# of animals** | **Reason** | **Treatment** | **Amount** |  |
| --- | --- | --- | --- | --- | --- |
| 2004-15 | | 35 | Antibacterial | Covexin 8 | 5 ml each |
| 2004-15 | | 35 | Respiratory | Viralshield 6 | 5 ml each |
| 2004-15 | | 35 | Deworm | Ivomec, Dectomax, or Normectin | 1 ml/33 kg |
| 3/21/16 | | 2 | Ringworm | Fungicide | Topical-amount varies |
| 3/23/16 | | 2 | Ringworm | Fungicide | Topical-amount varies |
| 6/1/16 | | 4 | Pinkeye | Noromycin 300 | 6.6 ml/100 kg |
| 6/1/16 | | 2 | Pinkeye | Noromycin 300 | 6.6 ml/100 kg |
| 6/1/16 | | 32 | Parasites | Cydectin | 1 ml/10 kg |
| 6/3/16 | | 4 | Pinkeye | Noromycin 300 | 6.6 ml/100 kg |
| 6/6/16 | | 4 | Pinkeye | Draxxin | 9 ml each |
| 6/14/16 | | 4 | Pinkeye | Noromycin 300 | 24 ml each |
| 6/20/16 | | 1 | Pinkeye | Duramycin | 46 ml each |
| 6/22/16 | | 1 | Pinkeye | Duramycin | 46 ml each |
| 6/24/16 | | 3 | Pinkeye | Duramycin | 45 ml each |
| 6/27/16 | | 3 | Pinkeye | Liquamycin | 45, 36, 45 ml |
| 6/27/16 | | 3 | Pinkeye | Liquamycin | 45, 36, 36 ml |
| 6/30/16 | | 3 | Pinkeye | Liquamycin | 45, 36, 36 ml |
| 7/5/16 | | 4 | Pinkeye | Penicillin | 1 ml each |
| 7/8/16 | | 4 | Pinkeye | Penicillin | 1 ml each |
| 7/11/16 | | 1 | Pinkeye | Oxytetracycline | 40 ml each |
| 8/31/16 | | 2 | Pinkeye | LA 200 | 40 ml each |
| 9/19/16 | | 5 | Pinkeye | LA 200 | 40 ml each |
| Fall ‘16 | | 35 | Blackleg prevention | 7-way Clostridial | 5 ml each |
| Fall ‘16 | | 35 | Respiratory | Viralshield 6 | 5 ml each |
| Fall ‘16 | | 35 | Pinkeye | Noromycin 300 | 6.6 ml/100 kg |
| 1/25/17 | | 35 | Deworm | SafeGuard | 1 kg/1000 kg body weight |
| 4/18/17 | | 35 | Deworm | Cydectin | 1 ml/10 kg body weight |
| 6/23/17 | | 35 | Deworm/Fly control | Ivermectin | 1 ml/10 kg body weight |
